# Corticosteroid or placebo injection combined with deep transverse friction massage, Mills manipulation, stretching and eccentric exercise for acute lateral epicondylitis: a randomised, controlled trial

**DOI:** 10.1186/s12891-015-0582-6

**Published:** 2015-05-20

**Authors:** Morten Olaussen, Øystein Holmedal, Ibrahimu Mdala, Søren Brage, Morten Lindbæk

**Affiliations:** Department of General Practice, Institute of Health and Society, University of Oslo, Kirkeveien 166, 0450 Oslo, Norway; Research section, Directorate for Labour and Welfare, Oslo, Norway

## Abstract

**Background:**

Lateral epicondylitis of the elbow is a frequent condition with long-lasting symptoms. Corticosteroid injection is increasingly discouraged and there is little knowledge on the combined effect of corticosteroid injection and physiotherapy for acute conditions. We wanted to investigate the efficacy of physiotherapy alone and combined with corticosteroid injection for acute lateral epicondylitis.

**Methods:**

A randomized, controlled study with one-year follow-up was conducted in a general practice setting in Sarpsborg, Norway. We included 177 men and women aged 18 to 70 with clinically diagnosed lateral epicondylitis of recent onset (2 weeks to 3 months). They were randomly assigned to one of three treatments: physiotherapy with two corticosteroid injections, physiotherapy with two placebo injections or wait-and-see (control). Physiotherapy consisted of deep transverse friction massage, Mills manipulation, stretching, and eccentric exercises. We used double blind injection of corticosteroid and single blind assessments. The main outcome measure was treatment success defined as patients rating themselves completely recovered or much better on a six-point scale.

**Results:**

One hundred fifty-seven patients (89 %) completed the trial. Placebo injection with physiotherapy showed no significant difference compared to control or to corticosteroid injection with physiotherapy at any follow-up. Corticosteroid injection with physiotherapy had a 10.6 times larger odds for success at six weeks (odds ratio 10.60, p < 0.01) compared to control (NNT = 3, 99 % CI 1.5 to 4.2). At 12 weeks there was no significant difference between these groups, but at 26 weeks the odds for success were 91 % lower (OR 0.09, p < 0.01) compared to control, showing a large negative effect (NNT = 5, 99 % CI 2.1 to 67.4). At 52 weeks there was no significant difference. Both control and placebo injection with physiotherapy showed a gradual increase in success.

**Conclusions:**

Acute lateral epicondylitis is a self-limiting condition where 3/4 of patients recover within 52 weeks. Physiotherapy with deep transverse friction massage, Mills manipulation, stretching, and eccentric exercises showed no clear benefit, and corticosteroid injection gave no added effect. Corticosteroid injections combined with physiotherapy might be considered for patients needing a quick improvement, but intermediate (12 to 26 weeks) worsening of symptoms makes the treatment difficult to recommend.

**Trial registration:**

ClinicalTrials.gov Identifier: NCT00826462

## Background

Lateral epicondylitis of the elbow (tennis elbow) is frequently encountered in general practice with an incidence of 5.5 per 1000 person-years [1]. It is characterised by pain and tenderness over the lateral humeral epicondyle and pain on resisted dorsiflexion and radial deviation of the wrist. It is usually a self-limiting condition, resolving in six to 12 months regardless of treatment, but symptoms may last up to two years or longer [2].

Most authors attribute the condition to a lesion in the short radial extensor muscle [3, 4]. The aetiology of this tendinopathy is probably degenerative rather than inflammatory [5, 6], and the term ‘lateral epicondylalgia’ has been proposed [7]. Patients with lateral epicondylitis are usually treated in general practice, but there is no consensus on which of many treatments is most effective. Corticosteroid injections are increasingly discouraged because of recurrence despite showing a marked short-term effect [8–10]. Physiotherapy with eccentric exercise and specific manual therapy (‘Manipulation with Movement’) show promising results [8, 10, 11]. The effect of combining physiotherapy with corticosteroid injection has been investigated in three studies with different duration of symptoms and length of follow-up [11–13]. None of these studies showed any additional benefit of physiotherapy compared with corticosteroid injection alone. Different physiotherapeutic modalities were used in these studies.

We have found no randomised, controlled studies conducted in general practice with long term follow-up that have looked exclusively at acute conditions. We wanted to investigate the effect of treatment on acute conditions. Considering the aetiology of tendinopathies as a continuum from physiology to pathology [5, 14], there might be less degeneration present in the tendon in acute stages with less permanent change in collagen structure, which might influence the effect of treatment. In general practice patients may have less severe or shorter duration of symptoms than at outpatient clinics, possibly giving a different study population and different effect of treatment.

For acute lateral epicondylitis in such a setting, we wanted to investigate the short- and long-term effect of physical treatment, which has been recommended [15]. We also wanted to see whether addition of corticosteroid would show any benefit, and if the well-known initial positive effect of corticosteroid injection [16, 17] would be longer lasting when combined with physical treatment.

We used two treatment groups. One group received corticosteroid injections and physical treatment, while the other received placebo injections and physical treatment. The placebo injections were assumed to have no effect, but were included for blinding purposes. We also included a control group receiving no treatment except NSAIDs. The first two groups enabled us to compare the treatment effect of corticosteroid injection to physical treatment alone. The control group enabled us to compare the effect of corticosteroid injection with physical treatment and physical treatment alone to a group representing the natural course of acute lateral epicondylitis. We chose a treatment protocol comprising well-known treatments based on earlier studies [8, 17], and our focus was on commonly used treatments more than investigating specific treatment modalities. At the time of planning the protocol (2008), there were no established standard physical treatment, but eccentric training was included based on promising results [8, 17, 18]. For practical and pragmatic reasons, we chose not to include ultrasound or other electrotherapeutic modalities.

### Objectives

The objective of this study was to investigate the clinical short- and long-term efficacy of corticosteroid injection or placebo injection with a combination of physical treatments for acute lateral epicondylitis in a primary care setting compared with a control group only treated with NSAIDs.

## Methods

### Trial design and setting

A randomized, placebo-controlled trial was conducted in a primary care setting in the city of Sarpsborg, Norway, as outlined in the published protocol [19]. The trial was approved by The Regional Committees for Medical Research Ethics - South East Norway, Faculty of Medicine, University of Oslo, The Norwegian Social Science Data Services and The Norwegian Medicines Agency, and all patients gave written informed consent.

### Participants

Patients aged 18–70 years seeing their general practitioner with pain of recent onset from the lateral part of the elbow were eligible for inclusion. Further inclusion criteria were pain increase on resisted dorsiflexion of the wrist with the elbow extended and the fingers flexed, resisted radial deviation of the wrist, or resisted extension of the third finger.

We defined acute lateral epicondylitis as symptom duration less than 3 months. To avoid light, self-limiting conditions, we pragmatically chose to exclude patients with less than 2 weeks symptom duration. We also excluded patients with tenderness within the muscle body itself (Cyriax type IV) [20] to avoid misdiagnosis (e.g., nerve entrapment) and focus the treatment with manipulation and injection on the tendon origin. Other exclusion criteria were treatment with corticosteroid injection or physiotherapy within the last 12 months, bilateral symptoms, previous surgical treatment for lateral epicondylitis, deformities of the elbow, cervical radiculopathy, referred pain from neck or shoulder, previous fractures or tendon ruptures in the elbow, systemic musculoskeletal disease, previous allergic reactions or contraindications to corticosteroids, lidocaine, or NSAIDs, pregnancy or breast-feeding, fertile females not on effective birth control, and psycho-social or other reasons for not being able to participate throughout the study.

### Interventions

In a six week treatment period, patients received one of three treatments: physiotherapy with two corticosteroid injections and naproxen orally, physiotherapy with two placebo injections and naproxen, or a wait-and-see treatment with naproxen.

#### Physiotherapy

A cooperating physiotherapist treated patients twice weekly for six weeks with deep transverse friction massage at the tendon origin for 15 minutes, Mills manipulation [21, 22] once each treatment session, and soft tissue treatment with stretching of the radial wrist extensors. The patients received oral and written instructions for home exercises daily for six weeks with eccentric exercise (three times 30 repetitions) and isolated stretching of radial wrist extensors (three times daily for 40 seconds). The eccentric exercise was done within tolerable pain level during the exercise by using a 500 ml bottle filled with increasing volume of water or sand as patients improved.

#### Corticosteroid injection

At start and after three weeks, patients received an injection with 10 mg triamcinolone acetonide (1 ml of 10 mg/ml) or placebo (1 ml of 0.9 % isotonic saline) and 0.5 ml of 2 % lidocaine. With the patient in a supine position, the elbow flexed, and the wrist pronated, the point of maximum tenderness was located. The needle was inserted at 90° down to the level of bone and then pulled back 1–2 mm, leaving several small depots at the surface of the tendon. The patient was informed of possible adverse effects and advised to avoid pain-provoking activities for the rest of the day. The second injection was not given if there had been adverse reactions or increase in symptoms.

#### General treatment and information

All groups received naproxen 500 mg twice daily for two weeks for pragmatic reasons, since the control group thus would receive some form of treatment in the initial six-week period. Paracetamol could be taken at the patient’s own discretion up to 4 grams daily and its use was recorded. No self-registration of medication was used. General advice was given to all groups, including the natural course of the condition and expected duration of symptoms. Patients were encouraged to use their arm as usual, but avoid pain-provoking activities. Additional treatment after the six-week treatment period and sick leave certification was given at the discretion of the general practitioner.

### Outcomes

A number of baseline characteristics were registered. The primary outcome measures were the patients’ evaluations of improvement after six, 12, 26, and 52 weeks [8]. On a six point Likert scale (much worse-worse-a little worse-some improvement-much improvement-completely recovered), a rating of much improvement or completely recovered was defined as treatment success [8, 11].

Secondary outcome measures included elbow pain, affected function and overall complaint, registered on a 100 mm Visual Analogue Scale (VAS). Pain-free and maximum grip strength was registered with a hand-held, analogue dynamometer as a mean of three measurements in a ratio of affected to unaffected side (Jamar Hydraulic Hand Dynamometer-5030 J) [8, 23, 24]. Pain on resisted dorsiflexion of the wrist and third finger was registered on a three-point scale (none, some, definite) [16, 17] and pain on eight every-day activities was registered using the Pain Free Function Index [25, 26]. Patients were asked at each follow-up after specific adverse events (increased pain in elbow, heart burn, dyspepsia, reflux symptoms, abdominal pain, gastritis, and ulcer) and checked for skin atrophy. They were also asked about the need for additional treatment and duration of sick leave.

### Sample size

Sample size was based on the ability to detect a 25 % difference in the success rate at three months. Earlier studies have shown a large success rate of corticosteroid injections for the first three months [8, 17]. Later, the success rate increases regardless of intervention. Based on earlier studies [17], we assumed a success rate of 55 % at three months in the least successful group. The target sample size was estimated at 52 patients per group (two tailed α: 0.05, β: 0.20) giving a total of 156 patients. With a loss to follow-up of 10 % and allocation in three groups, we decided to include 180 patients. Since the drop-out rate stayed near the 10 % prediction (20 drop-outs, 11 %), we decided to stop the inclusion at 177 patients.

### Randomisation and blinding

A computerised randomisation schedule was prepared by an independent researcher (ML), using numeric block randomisation with variable block size. Stratification of patients was not done. The patients were first assessed by one of two trial doctors. If inclusion criteriae were met, the patient was enrolled in the study. Only then was an independent research assistant contacted, who, by consulting the previously prepared randomisation schedule, allocated the patient to one of three treatments ensuring concealed allocation. The trial doctor then proceeded to do the baseline registrations and start the allocated treatment. A research assistant prepared the syringes used for the injection treatment and concealed its content by an opaque adhesive patch, thus blinding the content of the injection for both administering doctor and patient. To ensure blinded assessment of treatment effect, in the follow-up period from week six patients saw the other trial doctor, who was blinded for treatment. Patients were cautioned at each assessment not to disclose their treatment, and the success of blinding was assessed at 52 weeks by the trial doctor guessing which treatment the patient had received.

### Statistical methods and analysis

The statistical analysis was performed blinded to treatment group on an intention-to-treat basis using SPSS version 21 and Stata version 13. The percentage of treatment success was presented unadjusted, calculated based on the number of patients included, assuming those lost to follow-up had no success as outcome. Numbers needed to treat (NNT) was calculated using this unadjusted difference in success between groups at each follow-up.

Since a number of variables may have influenced results, we adjusted for these by using a regression model, with the resulting odds ratio giving an often different but more correct picture of the effect of the interventions. Our data consisted of both between-group observations and longitudinal data with observations at five different time points (baseline, six, 12, 26 and 52 weeks). Such observations within a subject tend to be correlated, and we therefore used the Generalized Estimating Equations (GEE) model to account for possible correlation between repeated measurements over time [27]. Continuous missing data was handled by assuming data was missing completely at random (MCAR) in the GEE models.

Univariate GEE logistic regression models were fitted to our primary outcome (treatment success) to identify significant covariates. The significant covariates at p < 0.05 were: to be in paid work, presumed cause of condition being overuse of the arm or hand in usual activities, pain score, affected function score, overall complaints score, and maximum grip strength ratio, all measured at baseline. Only these covariates were used in our final models to adjust for the effect of treatment over time on the binary responses (success of treatment and pain-free extension of wrist and third finger). For the continuous responses (pain, affected function, and overall complaints on VAS scales, grip strength ratios and Pain Free Function Index), we used a linear GEE regression model adjusted for the same covariates.

We performed statistical tests on treatment effectiveness at several time points. To reduce problems of multiple testing, we used a significance level of 1 % in the GEE models and computed the 99 % confidence intervals, as other authors have done [8, 11]. P-values are given in the tables. Differences between groups regarding adverse events, use of additional treatments, and sick leave were analysed at one time-point only (week 52). We used a Chi-square test for independence with a 5 % significance level. A one-way between-groups analysis of variance was conducted to compare differences in sick leave duration.

## Results

### Recruitment and participant flow

**Fig. 1:**
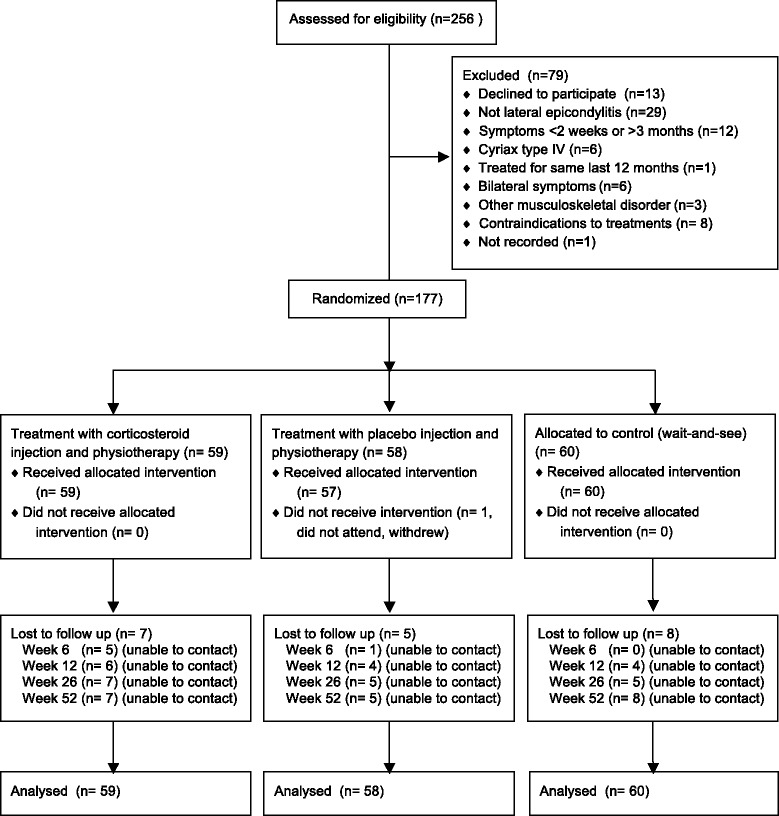
Study flow diagram showing recruitment, randomisation to treatment groups, and follow up rates

Two hundred fifty-six patients were referred to the study between April 2009 and June 2012. Of these, 79 were excluded; 69 did not meet the inclusion criteria and 10 declined to participate. A total of 177 patients were included in the study. The trial was completed in June 2013, with 157 patients (89 %) completing the primary outcome at 52 weeks follow-up. This met the minimum number required from the sample size calculations. Figure 1 summarizes the patient flow. 117 patients were randomized to treatment with physiotherapy. One patient in the placebo injection with physiotherapy group withdrew from the study before receiving any treatment. 103 (89 %) of the remaining 116 patients concluded the treatment in accordance with the physiotherapy protocol as assessed by the treating physiotherapist (having attended at least 8 of 12 sessions), 53 (91 %) in the placebo injection with physiotherapy group and 50 (85 %) in the corticosteroid injection (CI) with physiotherapy group. All patients in the injection groups received two injections except one patient in the placebo injection with physiotherapy group, who received only one injection.

### Baseline data

Before start, baseline characteristics were registered (Table 1). The groups were similar in regards to gender, age, duration and overall severity of symptoms, grip-strength and other characteristics.

**Table 1:** Baseline characteristics. Baseline characteristics of participants according to treatment groups and total study population. Values are numbers (percentages) unless otherwise stated

		Control	Placebo injection with physiotherapy	Corticosteroid injection with physiotherapy	Total
		(n = 60)	(n = 58)	(n = 59)	(N = 177)
Age	years, mean (SD)	44.0 (9.7)	48.8 (9.4)	47.9 (9.6)	46.9 (9.7)
Women		25 (42)	20 (35)	26 (44)	71 (40)
Higher education*		15 (25)	24 (41)	13 (22)	52 (29)
Exercises regularly		30 (50)	32 (55)	24 (41)	86 (49)
Paid work		53 (88)	49 (85)	52 (88)	154 (87)
Paid work		53 (88)	49 (85)	52 (88)	154 (87)
Manual labor		36 (60)	29 (50)	35 (59)	100 (57)
On paid sick-leave now**		14 (26)	14 (29)	23 (44)	51 (33)
Duration of complaints	(weeks, median (IQR))	7.5 (4‐10)	8 (4‐10)	6 (4‐10)	8 (4‐10)
Dominant elbow affected		41 (68)	42 (72)	43 (73)	126 (71)
Pain every day past week		58 (97)	55 (95)	57 (97)	170 (96)
Use of analgesics past week		15 (25)	15 (26)	25 (42)	55 (31)
Acute start of symptoms		42 (70)	25 (43)	27 (46)	94 (53)
Similar complaints earlier ^++^		16 (27)	9 (16)	16 (27)	41 (23)
Presumed cause					
overuse, usual activitities		34 (57)	38 (66)	38 (64)	110 (62)
overuse, unusual activities		25 (42)	19 (33)	21 (36)	65 (37)
Patient’s preference for treatment					
Physiotherapy		17 (28)	26 (45)	24 (41)	67 (38)
Injections		15 (25)	6 (10)	19 (32)	40 (23)
Wait and see		6 (10)	3 (5)	2 (3)	11 (6)
No preference		22 (37)	22 (38)	14 (24)	58 (33)
Pain Score on VAS (0‐100 mm)	mean (sd)	48 (21.5)	53 (19.3)	56 (19.7)	52 (20.4)
Affected Function on VAS (0: not affected, 100: very much affected)	mean (sd)	53 (21.8)	54 (22.5)	53 (21.7)	53 (21.9)
Overall Complaints on VAS (0: no complaints, 100: very large complaints)	mean (sd)	62 (20.5)	62 (19.3)	69 (17.1)	64 (19.2)
Pain-free grip strength ratio***	mean (sd)	35 (27)	38 (27)	37 (27)	36 (27)
Maximum grip strength ratio***	mean (sd)	72 (29)	77 (42)	70 (28)	73 (34)
Pain Free Function Index****	mean (sd)	5.4 (1.6)	5.3 (1.8)	5.5 (1.7)	5.4 (1.7)
Pain-free isometric dorsiflexion of wrist^+^		0/60 (0)	0/58 (0)	2/59 (3)	2/177 (3)
Pain-free isometric extension of third finger^+^		8/59 (14)	2/58 (3)	4/59 (7)	14/176 (8)

*: university/college

**: percentage of those with paid work

***: ratio affected/unaffected arm (x 100)

****: 0: full function, 8: no function

+: score 1 on 3 point Likert scale (1-no pain, 2-some pain, 3‐strong pain)

++: at least 3 months prior to current episode

### Outcomes and estimations

#### Primary outcome

**Fig. 2:**
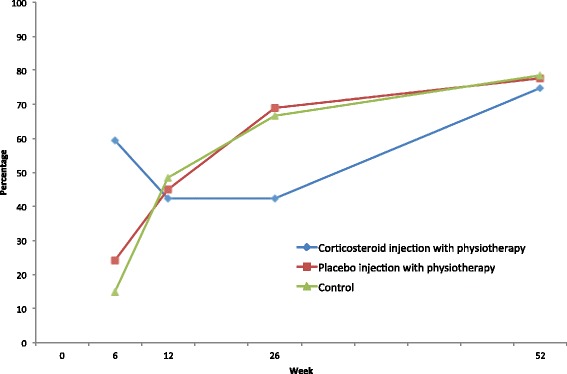
Unadjusted percentage of success at each follow up, defined as participants rating themselves much improved or completely recovered on a six-point scale

Unadjusted event rates of treatment success, defined as participants rating themselves much improved or completely recovered on a six-point scale, are shown in Fig. 2 and Table 2. The control group and placebo injection with physiotherapy group showed a gradual and very similar pattern of improvement, while the CI with physiotherapy group showed a marked improvement at six weeks, but then a lower rate of success at 12 and 26 weeks. At 52 weeks, the rates were similar across all groups. To compare groups statistically, estimated odds ratios for treatment success from the GEE regression model adjusted for significant covariates are given in Table 2. Placebo injection with physiotherapy showed no significant difference compared to control on any of the follow-ups. CI with physiotherapy had a 10.6 times larger odds for success at six weeks (odds ratio 10.60, p < 0.01) compared to control, suggesting a large initial treatment effect (NNT = 3, 99 % CI 1.5 to 4.2). At 12 weeks there was no significant difference between these groups, but at 26 weeks the odds for success were 91 % lower (OR 0.09, p < 0.01) compared to control, showing a large negative effect (NNT = 5, 99 % CI 2.1 to 67.4). At 52 weeks there was no significant difference. There was no significant difference between placebo injection with physiotherapy and CI with physiotherapy at any follow-up.

**Table 2:** Treatment success. Unadjusted event rates of treatment success, defined as participants rating themselves much improved or completely recovered on a six point scale; estimated odds ratio for success between groups using logistic generalized estimating equations (GEE) regression model adjusted for significant covariates with numbers needed to treat (NNT); estimated odds ratio for success within each group compared to week 6 using logistic GEE regression model adjusted for significant covariates; 99 % confidence intervals. NNT calculated from unadjusted treatment success values

Unadjusted percentage of treatment success (99 % CI) at each follow up									
	Control			Placebo injection with physiotherapy			Corticosteroid injection (CI) with physiotherapy		
Follow up	(n = 60)			(n = 58)			(n = 59)		
weeks	% (99 % CI)			% (99 % CI)			% (99 % CI)		
6	15 (3 to27)			24 (10 to 39)			59 (43–76)		
12	48 (32 to 65)			45 (28 to 62)			42 (26 to 59)		
26	67 (51 to 82)			69 (53 to 85)			42 (26 to 59)		
52	78 (65 to 92)			78 (64 to 92)			75 (60 to 89)		
Estimated Odds Ratio (99 % CI) and NNT ^+^ for treatment success between groups									
	Placebo injection with physiotherapy versus control	P	NNT (99 % CI)	CI with physiotherapy versus control	P	NNT (99 % CI)	CI with physiotherapy versus placebo injection with physiotherapy	P	NNT (99 % CI)
	OR > 1 favours first treatment in comparison								
6	3.37 (0.72 to 15.82)	0.04	11 **	10.60 (2.20 to 51.24)*	<0.01	3 (1.5 to 4.2)	3.14 (0.72 to 13.75)	0.045	3 (1.8 to 7.5)
12	0.18 (0.02 to 1.47)	0.04	29 **	0.15 (0.02 to 1.24)	0.02	17**	0.81 (0.10 to 6.37)	0.80	41 **
26	0.37 (0.04 to 3.35)	0.25	44 **	0.09 (0.01 to 0.76)*	<0.01	5 (2.1 to 67.4)	0.24 (0.03 to 2.05)	0.09	4 (2.0 to 26.3)
52	0.12 (0.01 to 1.50)	0.03	134 **	0.17 (0.01 to 2.63)	0.10	27 **	1.49 (0.13 to 17.12)	0.67	34 **
Estimated Odds Ratio (99 % CI) for treatment success compared to week 6 within each group									
	Control	P		Placebo injection with physiotherapy	P		CI with physiotherapy	P	
	OR > 1 indicates improvement compared to week 6								
12	7.17 (1.53 to 33.66)*	<0.01		1.30 (0.32 to 5.26)	0.63		1.05 (0.23 to 4.75)	0.93	
26	6.09 (1.27 to 29.29)*	<0.01		2.25 (0.47 to 10.81)	0.18		0.55 (0.13 to 2.39)	0.29	
52	14.84 (1.92 to 114.57)*	<0.01		1.70 (0.34 to 8.43)	0.39		2.54 (0.40 to 16.01)	0.19	

*: p < 0.01

**: the 99 % confidence interval for the absolute risk reduction extends from a negative number to a positive number, and a 99 % CI for the NNT cannot be computed

+: NNT calculated from unadjusted treatment success values

Looking at within group differences, there was a gradual increase in success over time for the control group, and the increased odds ratio for success was statistically significant at week 12, 26 and 52 compared to week six (Table 2). Placebo injection with physiotherapy also showed a gradual increase in success rate. For CI with physiotherapy, the percentage of success was high at six weeks, but then lower at 12 and 26 weeks.

#### Secondary outcomes

The GEE regression model showed overall no significant differences in favour of either placebo injection with physiotherapy or CI with physiotherapy compared to control for most of the secondary outcomes (Table 3). One exception from this overall picture was the estimated odds for pain-free isometric wrist extension that were 9.2 times higher for CI with physiotherapy compared to placebo injection with physiotherapy at six weeks (OR 9.2, p < 0.01). At 52 weeks, the result was reversed, with 94 % lower odds of pain-free wrist extension (OR 0.06, p < 0.01). Another exception at 26 weeks was isometric extension of the third finger, where CI with physiotherapy had 83 % lower odds of pain-free extension compared to control (OR 0.17, p < 0.01) and 84 % lower odds compared to placebo injection with physiotherapy (OR 0.16, p < 0.01). No other statistically significant differences were found between the treatment groups.

**Table 3:** Secondary outcomes. Mean (sd) scores and percentage (99 % CI) for secondary outcome measures at each follow up. Between group differences in estimated mean score using linear generalized estimating equations (GEE) regression model adjusted for significant covariates; between groups estimated odds ratio (OR) for no pain on two isometric movements using logistic GEE regression model adjusted for significant covariates; 99 % confidence intervals

Mean scores (sd) for each intervention					Difference in estimated mean score (99 % CI) between groups					
Outcome measure	Follow up	Control	Placebo injection with physiotherapy	Corticosteroid injection (CI) with physiotherapy	Placebo injection with physiotherapy versus control	P	CI with physiotherapy versus control	P	CI with physiotherapy versus placebo injection with physiotherapy	P
					Negative value favours first treatment in comparison					
Pain Score on VAS (0: no pain, 100: worst pain)										
	6 weeks	44 (25)	45 (23)	29 (25)	−3.7 (−12.4 to 5.0)	0.27	−3.2 (−12.0 to 5.6)	0.35	0.53 (−8.4 to 9.4)	0.88
	12 weeks	33 (26)	33 (26)	41 (26)	−3.7 (−12.5 to 5.1)	0.28	0.2 (−8.5 to 8.9)	0.95	3.9 (−4.9 to 12.7)	0.25
	26 weeks	19 (22)	21 (22)	38 (28)	−3.6 (−12.4 to 5.2)	0.29	1.8 (−7.0 to 10.6)	0.60	5.4 (−3.6 to 14.4)	0.12
	52 weeks	13 (19)	9 (11)	19 (23)	−6.0 (−14.9 to 2.9)	0.09	−2.9 (−11.8 to 6.0)	0.40	3.0 (−6.0 to 12.0)	0.38
Affected Function on VAS (0: not affected, 100: very much affected)										
	6 weeks	38(26)	45 (24)	25 (23)	8.3 (−1.8 to 18.4)	0.04	7.5 (−2.8 to 17.8)	0.06	−0.8 (−11.2 to 9.6)	0.85
	12 weeks	34 (28)	32 (25)	37 (29)	−1.2 (−11.5 to 9.0)	0.76	3.4 (−6.8 to 13.5)	0.40	4.6 (−5.7 to 14.9)	0.25
	26 weeks	16 (20)	17 (22)	28 (25)	0.29 (−10.0 to 10.6)	0.94	4.4 (−5.9 to 14.7)	0.27	4.1 (−6.4 to 14.6)	0.31
	52 weeks	10 (17)	9 (15)	16 (22)	0.24 (−10.2 to 10.7)	0.95	6.0 (−4.4 to 16.4)	0.14	5.8 (−4.7 to 16.2)	0.16
Overall Complaints on VAS (0: no complaints, 100: very large complaints)										
	6 weeks	51 (28)	50 (26)	32 (26)	−1.5 (−9.2 to 6.2)	0.62	−6.9 (−14.7 to 0.9)	0.02	−5.4 (−13.3 to 2.4)	0.08
	12 weeks	36 (27)	35 (28)	43 (30)	1.6 (−6.1 to 9.4)	0.59	−2.2 (−9.9 to 5.5)	0.47	−3.8 (−11.6 to 3.9)	0.21
	26 weeks	18 (20)	19 (23) 36 (26)	1.9 (−5.9 to 9.7)	0.52	−0.5 (−8.3 to 7.3)	0.86	−2.5 (−10.4 to 5.5)	0.42	
	52 weeks	12 (19)	9 (13)	20 (24)	2.5 (−5.4 to 10.3)	0.42	−1.0 (−8.9 to 6.9)	0.74	−3.5 (−11.4 to 4.5)	0.26
					Positive value favours first treatment in comparison					
Pain‐free grip strength ratio (x 100)^+^										
	6 weeks	57 (32)	50 (29)	58 (35)	6.1 (−15.9 to 28.1)	0.47	1.8 (−20.6 to 24.22)	0.84	−4.3 (−26.9 to 18.2)	0.62
	12 weeks	63 (36)	55 (25)	64 (43)	0.6 (−21.9 to 23.2)	0.94	−3.4 (−25.7 to 18.9)	0.70	−4.0 (−26.4 to 18.4)	0.65
	26 weeks	74 (37)	76 (27)	82 (48)	10.0 (−12.9 to 32.8)	0.26	3.4 (−19.4 to 26.2)	0.70	−6.6 (−29.8 to 16.6)	0.47
	52 weeks	91 (23)	90 (23)	100 (33)	11.8 (−11.7 to 35.4)	0.24	7.9 (−15.5 to 31.2)	0.39	−4.0 (−28.3 to 19.3)	0.66
Maximum grip strength ratio (x 100)^+^										
	6 weeks	74 (27)	80 (25)	87 (26)	1.7 (−14.6 to 18.0)	0.79	6.8 (−9.8 to 23.4)	0.29	5.1 (−11.6 to 21.8)	0.43
	12 weeks	89 (28)	88 (23)	83 (29)	−7.2 (−23.7 to 9.3)	0.26	−3.7 (−20.2 to 12.7)	0.57	3.6 (−13.0 to 20.1)	0.58
	26 weeks	99 (19)	99 (20)	89 (27)	−4.5 (−21.0 to 12.0)	0.48	−2.7 (−19.3 to 13.9)	0.67	1.8 (−15.1 to 18.6)	0.79
	52 weeks	104 (15)	102 (17)	96 (19)	−7.6 (−24.3 to 9.2)	0.25	−5.0 (−21.7 to 11.7)	0.44	2.6 (−14.3 to 19.4)	0.70
					Negative value favours first treatment in comparison					
Pain Free Function Index (0–8, 0: full function, 8: no function)										
	6 weeks	4.82 (2.16)	4.40 (2.01)	2.78 (2.22)	−0.13 (−1.20 to 0.94)	0.76	−0.52 (−1.60 to 0.57)	0.22	−0.39 (−1.49 to 0.71)	0.36
	12 weeks	3.37 (2.36)	3.62 (2.43)	3.42 (2.63)	0.38 (−0.70 to 1.46)	0.36	−0.15 (−1.22 to 0.92)	0.72	−0.54 (−1.62 to 0.55)	0.20
	26 weeks	2.00 (2.25)	1.83 (2.08)	2.97 (2.53)	−0.04 (−1.12 to 1.05)	0.93	0.46 (−0.63 to 1.55)	0.27	0.50 (−0.61 to 1.60)	0.24
	52 weeks	1.40 (1.90)	1.03 (1.67)	1.64 (2.04)	−0.18 (−1.28 to 0.92)	0.67	0.01 (−1.08 to 1.11)	0.98	0.20 (−0.91 to 1.30)	0.65
Percentage (99 % CI) for each intervention					Estimated Odds Ratio (99 % CI) between groups					
					OR > 1 favours first treatment in comparison					
No pain on three point Likert scale on dorsiflexion of wrist^++^										
	6 weeks	8 (−1 to 18)	3 (−3 to 10)	36 (20 to 52)	0.38 (0.04 to 3.96)	0.29	3.47 (0.71 to 17.1)	0.04	9.2 (1.09 to 77.74)*	<0.01
	12 weeks	17 (4 to 29)	12 (1 to 23)	22 (8 to 36)	1.07 (0.06 to 18.18)	0.95	0.66 (0.07 to 5.82)	0.63	0.62 (0.04 to 9.01)	0.65
	26 weeks	38 (22 to 54)	29 (14 to 45)	19 (6 to 32)	1.76 (0.13 to 24.65)	0.58	0.20 (0.02 to 1.63)	0.048	0.11 (0.01 to 1.45)	0.03
	52 weeks	50 (33 to 67)	60 (4 to 77)	36 (20 to 52)	3.88 (0.29 to 53.02)	0.18	0.23 (0.03 to 1.71)	0.06	0.06 (0.01 to 0.69)*	<0.01
No pain on three point Likert scale on isometric extension of third finger^++^										
	6 weeks	17 (4 to 29)	16 (3 to 28)	44 (27 to 61)	0.90 (0.22 to 3.70)	0.84	2.95 (0.81 to 10.72)	0.03	3.29 (0.88 to 12.27)	0.02
	12 weeks	22 (8 to 35)	24 (10 to 39)	36 (20 to 52)	1.21 (0.18 to 8.03)	0.80	1.14 (0.19 to 6.97)	0.86	0.94 (0.15 to 5.87)	0.93
	26 weeks	58 (42 to 75)	53 (37 to 70)	31 (15 to 46)	1.07 (0.17 to 6.61)	0.93	0.17 (0.03 to 0.96)*	<0.01	0.16 (0.03 to 0.93)*	<0.01
	52 weeks	63 (47 to 79)	76 (61 to 90)	53 (36 to 69)	2.04 (0.30 to 13.96)	0.34	0.33 (0.06 to 1.98)	0.11	0.16 (0.03 to 1.06)	0.012

^+^: ratio affected/unaffected arm

^++^: no pain, some pain, strong pain

*: p < 0.01

For both control and placebo injection with physiotherapy, there seemed to be a gradual within-group improvement over time across all secondary outcomes (Table 3). For CI with physiotherapy, there was a worsening across most outcomes at 12 and 26 weeks.

#### Sick leave and use of additional treatments

Twenty-three of patients in the control group used additional treatment and 52 % had been on paid sick leave, with a mean of 43 full days and 60 partial days on leave. Similar values were found for the two treatment groups, indicating that the condition considerably affects the function and activity of patients. There was no significant association between treatment and the use of paid sick leave during the follow-up (p = 0.88, Table 4). For those on paid-sick leave, a one-way analysis of variance showed no statistically significant differences in mean duration of sick leave for neither full (p = 0.25) nor partial (p = 0.07) sick-leave. There was no significant association between treatment and the number of patients receiving additional treatments in each group (p = 0.63). When we looked at each additional treatment, we found an association between treatment group and use of additional physiotherapy (p = 0.02). A post-hoc 2 × 2 comparison showed significantly more use of additional physiotherapy in the CI with physiotherapy group compared to the placebo injection with physiotherapy group (p < 0.01) (Table 4).

**Table 4:** Sick leave, additional treatments and adverse events. Use and length of paid sick leave, use of additional not per protocol treatments and reported adverse events for each treatment group. Values are numbers (percentages) if not otherwise stated. Chi-square test of independence and one-way analysis of variance for between groups association

	Control	Placebo injection with physiotherapy	Corticosteroid injection with physiotherapy	P
	(n = 60)	(n = 58)		Chi‐square test
Paid sick leave during 52 week follow‐up				
Number (percentage) on leave	30 (52)	31 (56)	30 (55)	0.88
Duration of leave for those on sick‐leave:				
Days on full leave mean (95 % CI)	43.6 (22.0 to 65.1)	73.2 (34.2 to 112.2)	47.1 (30.0 to 64.3)	0.25*
Days on partial leave mean (95 % CI)	60.0 (23.1 to 96.9)	22.0 (6.8 to 37.3)	29.2 (11.2 to 47.2)	0.07*
Additional treatments				
Number of patients receiving additional treatments	13 (23)	12 (22)	16 (30)	0.63
Type of treatment:				
Pain killers	6 (11)	4 (7)	2 (4)	0.37
NSAIDs perorally	7 (13)	9 (16)	11 (20)	0.54
topical NSAIDs	3 (5)	1 (2)	2 (4)	0.62
other medication	3 (6)	0 (0)	3 (6)	0.22
physiotherapy	4 (7)	1 (2)	9 (17)	0.02^+^
other	6 (11)	4 (7)	4 (8)	0.76
Adverse Events				
Patients reporting adverse events	9 (16)	5 (9)	6 (11)	0.51
Type of adverse events				
increased pain	2 (4)	2 (4)	0 (0)	0.37
GI side effects**	8 (15)	3 (6)	6 (11)	0.30
skin atrophy	0 (0)	0 (0)	0 (0)	‐

*: one‐way ANOVA

**: heart burn/ dyspepsia, reflux symptoms, abdominal pain, gastritis, ulcer

+: p < 0.05

The assessors correctly guessed treatment allocation in 47 % of the total cases. For CI with physiotherapy, correct treatment was guessed in 61 % of cases, for placebo injection with physiotherapy in 37 %, and for control in 44 % of cases. A Chi-square test for independence indicated significant association between treatment group and correctly guessing the treatment (p = 0.04). A post-hoc 2 × 2 comparison showed significant differences between CI with physiotherapy and placebo injection with physiotherapy (p = 0.01).

### Harms

There was no significant association between treatment group and the number of reported adverse events (p = 0.51) (Table 4). There were no serious adverse events. The most frequently reported was mild gastrointestinal side effects, which 15 % in the control group reported.

## Discussion

There were no significant differences between placebo injection with physiotherapy and control for any outcome at any follow-up. CI with physiotherapy showed significantly higher odds for success compared to the control group at six weeks, but at week 26 the effect was negative. At 52 weeks there was no significant difference. There was no statistical difference in odds ratio for success between placebo injection with physiotherapy and CI with physiotherapy at any follow-up. CI with physiotherapy showed higher odds for pain-free wrist extension compared to placebo injection with physiotherapy at six weeks, but at 52 weeks the effect was negative. For extension of the third finger, CI with physiotherapy had lower odds of pain-free extension than both control and placebo injection with physiotherapy at 26 weeks.

The absence of significant differences between CI with physiotherapy and placebo injection with physiotherapy show that there was no added effect of steroid injection to physiotherapy, and the pattern of effect seemed to follow the well-known trajectory of steroid injections. The negative effect of CI with physiotherapy on self-rated success at intermediate follow-up might be explained by the fact that patients rated themselves worse compared to the very good initial response. However, other outcomes did indicate that there also was an objective worsening at intermediate follow-up. The initially good response might also have led patients to return to normal activity too soon, and thus induce a recurrence of symptoms. The use of corticosteroid might have delayed the tendon repair process and thereby caused a worsening in the intermediate term follow-up. The steroid injections were well tolerated, but there was significantly more use of additional physiotherapy in the follow-up period in the CI with physiotherapy group compared to the placebo injection with physiotherapy group. This might indicate a negative effect of steroid injection.

CI with physiotherapy showed a large improvement in success rate at six weeks, but at 12 and 26 weeks it was lower than at six weeks. Patients in the control group showed a statistically significant increase in odds ratio of success over time and a gradual improvement across secondary outcomes. This large improvement would indicate that the condition itself improves considerably without active treatment, making it difficult to show significantly better results for any treatment.

In addition to results that were statistically significant with a chosen level of significance at 0.01, we also found effects with a level between 0.01 and 0.05. These findings may be considered clinically important even if they were not statistically significant at our chosen level of p < 0.01. There was a non-significant tendency towards a positive effect on treatment success for placebo injection with physiotherapy compared to control at six weeks (p = 0.04), but at 12 and 52 weeks, the tendency was negative (p = 0.04 and p = 0.03 respectively). At 12 weeks, we found a tendency towards a negative effect for CI with physiotherapy (NNT = 17, p = 0.02). There was a non-significant tendency in favour of CI with physiotherapy at six weeks and in favour of placebo injection with physiotherapy at 26 weeks. For some secondary outcomes, there were significant differences at p < 0.05 between groups at a few time-points (Table 3). Placebo injection with physiotherapy was worse than control at 6 weeks for affected function on VAS, CI with physiotherapy was better than control at 6 weeks for overall complaints on VAS, pain free dorsiflexion of wrist and isometric extension of third finger. At 26 weeks, CI with physiotherapy was worse than control for pain-free wrist dorsiflexion. CI with physiotherapy was worse than placebo injection with physiotherapy at 26 weeks for pain-free dorsiflexion of wrist and extension of third finger, but better for isometric extension of third finger at 6 weeks.

### Strengths and weaknesses

We used a 52-week follow-up with several assessments to register the development of outcomes over the natural course of the condition. The general practice setting and the choice of ordinary, well known physiotherapeutic treatment modalities would ensure good external validity. Double blind injections and blinded assessors were also strengths of the study. There were few dropouts and the adherence to treatment protocol was good. There were similar results across several outcomes. We chose to use statistical models that would minimize Type-1 errors, since this is a non-serious, self-limiting condition. We also chose to take into account multiple testing and non-independence of data across several follow-ups.

Strict inclusion criteria, especially the exclusion of patients who had recently received other treatment, limit the generalizability of our results. The patients were not blinded to the physiotherapy treatment, and that may have influenced outcome. We did not log the home exercises and the use of only one physiotherapist limited the generalizability. A strict physiotherapy protocol prohibited an individual adjustment of treatment, and may have influenced results. The long follow-up made it difficult to avoid use of not-per-protocol treatments. Painkillers and additional treatment may have influenced results, but we found no differences in use between the groups except for additional physiotherapy in the CI with physiotherapy group. We did not look specifically at recurrence rates, which might have given more information for the group that received corticosteroid injection.

### Differences to other studies

Our findings were in contrast to other recently published studies [8, 11] and systematic reviews [9, 10, 15], which have found a short-time effect of physiotherapy [11]. They discourage the use of corticosteroid injection due to high recurrence rates and a possible negative effect at 52 weeks [8, 11]. There were important differences between these studies and ours; we included only acute conditions and had a general practice setting. In Coombes’ [11] and Bisset’s [8] studies, the duration of symptoms were longer (16 and 22 weeks median respectively), indicating a more chronic condition. On the other hand, a study in general practice found that corticosteroid injection gave a very good initial response and no worse results later on, although 1/3 of patients had duration of symptoms longer than 3 months [16]. Earlier studies investigating the combination of physiotherapy and injection have found no added benefit, Coombes for more chronic conditions [11], others with shorter follow-up [12, 13]. Our results agree with this, and found no added effect of steroid injection. A third difference to earlier studies was the different physiotherapeutic modalities used. Coombes and Bisset used a multi-dimentional exercise program (‘Mobilization with Movement’) that included isometric, concentric and eccentric exercise. The eccentric exercise used in our study was low load with 0.5–1 kg and 3 × 30 repetitions. Our findings suggest that physiotherapy with eccentric exercise might be less useful for acute conditions.

Tendinopathy is a continuum of changing conditions [14], requiring different treatments at different stages. Looking at the primary outcome of success, there seems to be reasonable homogeneity between the control group in our study and the placebo injection group in Coombes’ study [11], suggesting that the duration of symptoms and study setting did not affect the outcome for these groups. The largest difference in success between these two studies appears to be for the physiotherapy with placebo groups, suggesting that the difference in the physical treatments influenced the outcome more than setting and duration of symptoms. Treatment with steroid injection combined with physical treatment on acute lateral epicondylitis seems to follow the well-known pattern of short-term benefit and intermediate recurrence. Our long-term results showed no worse effect than control or physical treatment with placebo injection. Compared to other published results [8, 11], the evidence for long-term ramifications of this treatment still seems uncertain.

Although much research has been done on corticosteroid injection alone, it would be interesting to study the efficacy on acute conditions, using similar inclusion criteria as in our study. The possible need for different treatments at different stages in the course of the conditions would be useful to investigate further, including the possible difference in effect of physiotherapy on acute and chronic conditions.

## Conclusions and implications

Our results suggest that acute lateral epicondylitis is a self-limiting condition where 3/4 of the patients recover within 52 weeks without active treatment, but 1/4 had long-lasting symptoms and the use of sick-leave and additional treatments was high. Placebo injection with physiotherapy consisting of deep transverse friction massage, Mills manipulation, stretching, and eccentric exercises showed no clear beneficial effect on acute lateral epicondylitis. For CI with physiotherapy, we found a large positive effect on success of treatment at six weeks, no difference at 12 weeks, worsening at 26 weeks but no significant negative effect at 52 weeks compared to control. We found no significant differences between CI with physiotherapy and placebo injection with physiotherapy, suggesting no added effect of steroid injection. Secondary outcome measures gave similar results.

For patients needing a quick improvement, corticosteroid injections combined with the physiotherapeutic treatment used in this study might be considered, but they should be informed of a likely intermediate worsening of symptoms. This tendency makes the treatment difficult to recommend in general, even if we found no difference between groups at 52 weeks.
